# Aortopulmonary fistula as a rare complication of bioprosthetic aortic valve endocarditis: a case report

**DOI:** 10.1093/ehjcr/ytaf659

**Published:** 2025-12-18

**Authors:** Thomas Saliba, David Rotzinger, Mariama Touray, Guillaume Fahrni

**Affiliations:** Radiology Department, Centre Hospitalier Universitaire Vaudois, Rue du Bugnon 46, Lausanne 1005, Switzerland; Radiology Department, Centre Hospitalier Universitaire Vaudois, Rue du Bugnon 46, Lausanne 1005, Switzerland; Cardiology Department, Centre Hospitalier Universitaire Vaudois, Rue du Bugnon 46, Lausanne 1005, Switzerland; Radiology Department, Centre Hospitalier Universitaire Vaudois, Rue du Bugnon 46, Lausanne 1005, Switzerland

## Case description

A 67-year-old man presented for the management of a *Staphylococcus lugdunensis* bioprosthetic valve endocarditis (PVE).

**Figure 1:**
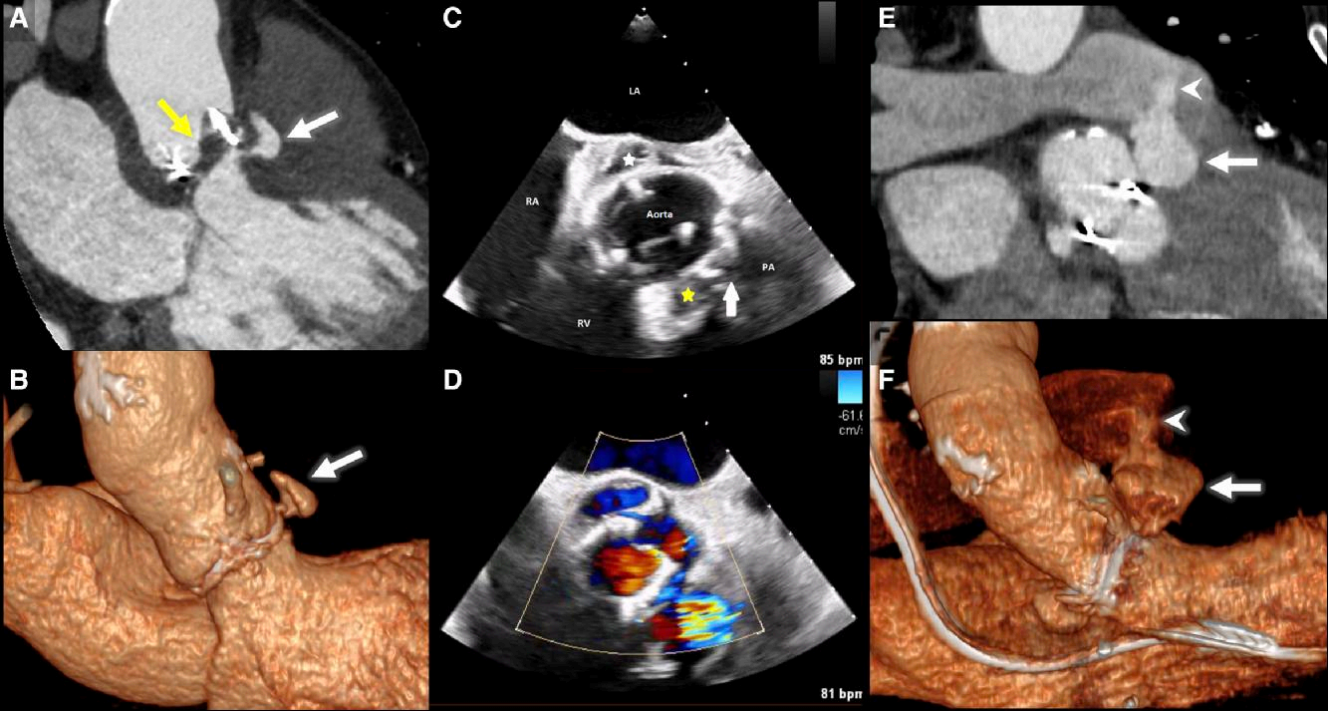
Imaging of prosthetic valve endocarditis complicated by pseudoaneurysm and aortopulmonary fistula. Contrast-enhanced CT scan (CT angiography) (*A*) and 3D virtual reconstruction (3D VR) (*B*) showing hypoattenuated leaflet thickening (yellow arrow) and a contained pseudoaneurysm (white arrow) upon admission. Cardiac transoesophageal echocardiogram in B-mode (*C*) and Doppler mode (*D*) of the aortic valve acquired post-operatively, revealing pseudoaneurysm (yellow star) with evidence of fistulization to the pulmonary trunk (white arrow). A second, smaller pseudoaneurysm is also visible on the opposite side of the valve (white star). Postoperative CT angiography (*E*) and 3D virtual reconstruction (*F*) showing rapid progression in size of the pseudoaneurysm (white arrow) with new-onset fistula to the pulmonary trunk (white arrowhead). LA = left atrium, PA = pulmonary artery, RA = right atrium, RV = right ventricle.

An ECG-gated cardiac CT angiography (CTA) revealed prosthetic valve degeneration with hypoattenuated leaflet thickening (HALT), complicated by a pseudoaneurysm of the left cusp (*Figure 1A and B*), resulting in its emergent surgical replacement.

The surgeons discovered vegetations on both the aortic and ventricular surfaces of the prosthetic valve, alongside an annular abscess extending towards the non-coronary commissure, creating a 1-cm cavity between the left and right commissures. The patient underwent debridement of the abscess with resection and repair of the pseudoaneurysm alongside the valvular replacement.

A post-operative transoesophageal echocardiogram (TEE) revealed a pseudoaneurysm of the prosthetic valve, raising suspicion of a fistula between the pseudoaneurysm and the pulmonary artery (*Figure 1C and D*).

A cardiac CTA was performed the following day, 14 days after the aortic valve replacement, revealing rapid progression of the pseudoaneurysm of the new prosthetic valve and confirmed the presence of a newly formed fistula between the aortic valve and the pulmonary trunk (*Figure 1E and F*).

The patient passed away 3 days later, following complications of sepsis and strokes.

Aortopulmonary fistula is a rare but life-threatening complication of prosthetic valve endocarditis, associated with high mortality. When PVE occurs, it may lead to the formation of pseudoaneurysms, which are best detected with CTA or TEE.^1^ If a fistula is suspected, invasive coronary angiography should be avoided due to increased risks of adverse events.^2^

Once pseudoaneurysms develop, they can be further complicated by the formation of fistulas, which are perforations caused by erosion between two cavities.^1^ In such cases, TEE is more accurate than CTA due to its superior temporal resolution.^1^

This case underscores the importance of early multimodality imaging in PVE with persistent sepsis as complications such as aorto-pulmonary fistula may develop rapidly despite surgical intervention.

## Data Availability

No new data was generated or analysed in support of this research.
